# New Mode of Preparing Objects for the Microscope

**Published:** 1869-06

**Authors:** 


New Mode of Preparing Objects for the Microscope.—M. Rauvier proposes (Archives de Physiologie) a new and simple method, which consists in the employment of picric or carbazotic acid. This acid is only moderately soluble in water, and a saturated solution may therefore be employed. It possesses the further advantage of being very cheap. It is admirably adapted for all tissues containing much blood, and therefore for specimens of liver, lung, etc. It appears to act by effecting coagulation of the albuminous substances, though, unlike alcohol and chromic acid, it does not occasion any fusion of the constituents of the tissue. The red globules retain their form and characters extremely well. The portion of tissues required to be examined should be plunged into the solution, and after the lapse of twenty-four hours it will be found to have acquired sufficient firmness to permit of very fine sections being made with a razor. The saving of time by this method as compared with the chromic acid is immense. The preparations will take color from carminate of ammonia, and may be preserved in glycerine.—*Lancet, Nov.* 21, 1868.—*Med. News and Library.*

